# Prevalence and risk factors of chronic oral complications in head and neck cancer therapies: A retrospective study

**DOI:** 10.4317/medoral.26823

**Published:** 2024-10-13

**Authors:** Eloy Benito-Ramal, Alex Camacho-Mourelo, Beatriz González-Navarro, José López López, Enric Jané-Salas

**Affiliations:** 1 Dental Student. School of Medicine and Health Sciences, Campus de Bellvitge, Universitat de Barcelona, Spain; 2 ORCID 0000-0002-5951-7499. Department of Odontostomatology. Faculty of Medicine and Health Sciences (Dentistry), University of Barcelona; Oral Health and Masticatory System Group (Bellvitge Biomedical Research Institute) IDIBELL. University of Barcelona, Barcelona, Spain; 3 ORCID 0000-0001-8035-4412. Department of Odontostomatology. Faculty of Medicine and Health Sciences (Dentistry). University of Barcelona; Clinical Chief of Odontological Hospital University of Barcelona; University of Barcelona Oral Health and Masticatory System Group (Bellvitge Biomedical Research Institute) IDIBELL. Barcelona, Spain; 4 ORCID 0000-0002-3574-4603. Department of Odontostomatology. Faculty of Medicine and Health Sciences (Dentistry), University of Barcelona; Oral Health and Masticatory System Group (Bellvitge Biomedical Research Institute) IDIBELL. University of Barcelona, Barcelona, Spain

## Abstract

**Background:**

Oncological therapy can trigger various complications and side effects in certain tissues, such as the oral cavity, inducing a direct or indirect impact on basic functions and the patient's quality of life. The aim of the study is to determine the prevalence of chronic oral complications of oncological treatments in patients with head and neck cancer and assess their possible relationship with risk indicators associated with the patient, the tumor, and the treatment.

**Material and Methods:**

A retrospective, single-center, observational pilot cohort study was designed at the Dental Hospital of the University of Barcelona, involving patients with head and neck cancer who underwent surgery, non-surgical oncological therapy (radiotherapy/chemotherapy/immunotherapy), or combined therapy. Medical histories were analyzed, and data related to demographics, toxic, hygienic, and dietary habits, systemic and oral health status, characteristics of cancer and its treatment, and registered chronic oral complications were collected. The results were expressed in descriptive measures (means, standard deviations, counts, prevalence, and 95% confidence intervals), and for statistical associations, parametric and non-parametric tests were used.

**Results:**

The overall prevalence of chronic oral complications was 92.57%. Dental disease (81.14%), periodontal disease (65.14%), and hyposalivation/xerostomia (62.86%) showed the highest prevalence. Advanced age, certain cancer locations, advanced cancer stages, and oncological therapy including radiotherapy were significantly associated with the presence and number of complications.

**Conclusions:**

The elevated noticed prevalence necessitates rigorous monitoring and preventive care. The combination of risk factors can significantly contribute to oral complications. Understanding these factors services dentists establish protocols for preventing, diagnosing, and treating oncology patients.

** Key words:**Oral complications, oral side effects, toxicity, radiotherapy, chemotherapy, immunotherapy, risk factors.

## Introduction

Head and Neck cancer (HNC) is progressively increasing in incidence in our society, ranking seventh among the most common cancers globally. Approximately 660,000 new cases and 325,000 deaths are recorded annually, with a projected 30% annual increase by 2030. Approximately 90% of cases are squamous cell carcinomas (SCC), originating in the epithelium of the oral cavity, pharynx, and larynx. The risk of HNC increases with age, tobacco and alcohol consumption, sun exposure, malnutrition, physical trauma, and Human papillomavirus infection (HPV). The current trend has shifted towards an increase in affected young women, especially in European countries, due to HPV infection. Five-year survival stands at 50-70%, although it varies depending on geographical location, type and location of cancer, and stage at diagnosis (1,2).

HNC therapy is based on various modalities: surgery (CX), radiotherapy (RT), chemotherapy (QT), biological therapies, and immunotherapy (IT), which can be combined as needed. Treatment choice depends primarily on tumour location, stage, and grade. It is initially treated, whenever possible, with CX, followed by RT with or without concurrent QT (3). Despite being considerably effective, these treatments can cause side effects in certain body tissues, such as the oral cavity, directly or indirectly impacting basic functions such as chewing, salivation, swallowing, taste, speech, and nutrition. The gastrointestinal mucosa, including the oral mucosa, is susceptible due to its rapid cell turnover rate, complexity and diversity of the microflora, and trauma to oral tissues. Primary factors contributing to these effects include lethal and sublethal tissue damage, immune system suppression, and interference with the physiological healing process (4-6).

The frequency and type of oral complications vary depending on the oncological treatment. The Oral Care Study Group (OCSG) of the Multinational Association of Supportive Care in Cancer / International Society of Oral Oncology (MASCC / ISOO) (7) establishes that the most common alterations associated with RT and QT, according to current prevalence, are mucositis, infections, dysfunction of salivary glands and taste, and oral pain. Generally, patients treated with QT experience acute toxic effects, while those irradiated not only suffer from acute complications but also from chronic vascular, mucosal, and connective tissue lesions, salivary gland dysfunction, muscle, and bone damage leading to secondary complications such as dehydration, dysgeusia, dysphagia, malnutrition, xerostomia, dental caries, trismus, bleeding, neuropathies, soft tissue and bone necrosis, and mucosal, muscular, and cutaneous atrophy and fibrosis (8-16). Additionally, advancements in targeted biological therapies and IT, with the implementation of experimental protocols, have introduced clinicians to a new spectrum of toxicities (17).

In the literature, indicators of risk for oral complications could be grouped into those associated with the patient, cancer and the treatment itself. Some of these include advanced age, smoking, poor oral hygiene, deteriorated dental and periodontal health, immunosuppression, the type and intensity of RT, concurrent QT, and prior invasive dental treatments (4,8-16,18,19). Understanding these factors provides an opportunity to personalize treatment plans to optimize cancer care while minimizing associated toxicity. The elimination of pre-existing dental, periapical and periodontal infections, as well as the implementation of oral hygiene protocols and reduction of factors affecting oral mucosa integrity, can decrease the frequency and severity of oral complications (3). Precision medicine, along with preventive, palliative, and curative interventions, is essential in managing oral sequelae, and dentists should have the knowledge and tools to provide good oral health care.

The retrospective study aims to determine the prevalence of chronic oral complications of oncological treatments in HNC patients and to assess its association with risk indicators.

## Material and Methods

- Study design

A retrospective, single-center, observational pilot cohort study was conducted on HNC patients seen at the Dental Hospital of the University of Barcelona (HOUB) between 2014 and 2023. The guidelines established by STROBE were followed (20).

- Sample population and eligibility criteria

The inclusion criteria were patients over 18 years old with a history of HNC who had received single oncological therapy of CX, RT, QT, or IT, with or without combined therapy, and seen by dentists and professors from the Dentistry unit in oncologic and immunocompromised patients at HOUB. All patients with a different cancer, other oral mucosal pathologies, and without enough medical information were excluded.

The prevalence of professionally reported oral side effects of QT, IT, and RT, has been estimated between 31-93% (5,21), 15-25% (17), and 90-100% (5), respectively. Considering an infinite population, a confidence level of 90%, an absolute precision of 4%, and assuming that these percentages of the patients would suffer oral secondary effects, the required sample size for this study was 542 patients.

- Retrospective data collection

A single observer analyzed patients' medical records through the Gesden® G5 program. Patient data registration was anonymous and randomized. Data organization was carried out in an Excel document for subsequent statistical analysis based on the variables under study: age, gender, smoking, alcohol consumption, oral hygiene and nutrition, systemic pathology, active medication, dental and periodontal status, previous/posterior invasive dental treatments, cancer type and location, staging, oncological therapy, RT dose in Gray (Gy), and finally information regarding complications such as presence/absence of each, diagnostic timing, progression, and overall number of complications. The following were considered chronic complications: hyposalivation and xerostomia, taste dysfunction, dental and periodontal disease, oral pain, mucositis, trismus, mucosal atrophy and fibrosis, soft tissue necrosis, viral/bacterial/fungal infection, odynophagia, dysphagia, osteoradionecrosis (ORN) and osteochemonecrosis (OQN), and neuropathies. The information was collected whenever available; otherwise, it was considered non-valuable (NV). In the statistical analysis, the NV category was excluded and marked as missing values. Consequently, in some groups, the sample size was smaller than the total number of patients.

- Statistical analysis

For data analysis, descriptive and inferential statistics were applied. Descriptive analysis considered mean and standard deviations (SD) in quantitative variables, and counts, prevalence, and 95% confidence intervals (CI) in qualitative variables.

For inferential analysis, the study aimed to evaluate the association between risk factors and the prevalence and mean of complications. The chi-square test was applied to qualitative variables to examine prevalence, while Fisher's exact test was used for small, expected frequencies. The Student's t-test was used for mean complications in dichotomous variables after verifying normality and homogeneity. The Mann-Whitney U test was the non-parametric alternative. ANOVA compared mean complications across groups, with the Kruskal-Wallis H test for non-normal distributions. Post hoc tests with Bonferroni corrections were applied. Logistic regression assessed the independent effect of risk factors, studying the Odds Ratio (OR) to determine complication probabilities. Results were interpreted with a significance level of *p*<0.05 and a 95% CI, using SPSS v29.0.

## Results

- Sample description

Out of 534 evaluated patients, 359 were excluded. Of the 175 included, 59 were women (33.71%) and 116 were men (66.29%), with an average age of 62.96 years (SD = 11.56 and range 23-89). Non-smokers or ex-smokers constituted 46.10% (n = 71), smokers until diagnosis 40.26% (n = 62), and 13.64% (n = 21) were active smokers throughout therapy. Alcohol consumption was observed in 45.99% (n = 63), and poor oral hygiene in 90.32% (n = 56). Systemic pathology was present in 81.01% (n = 128), and 79.75% (n = 126) were on medication. Dental pathology was seen in 86.79%, with 66.04% (n = 35) having at least one carious lesion and 20.75% (n = 11) showed pulpal or periapical pathology. Gingivitis was perceived in 20% (n = 11) and 70.91% (n = 39) presented periodontal disease. Invasive dental treatments were performed in 47.75% (n = 44). 95.15% (n = 157) had SCC, mainly in the pharynx (27.71%) and tongue (22.29%) (Table 1).

**Figure 1:**
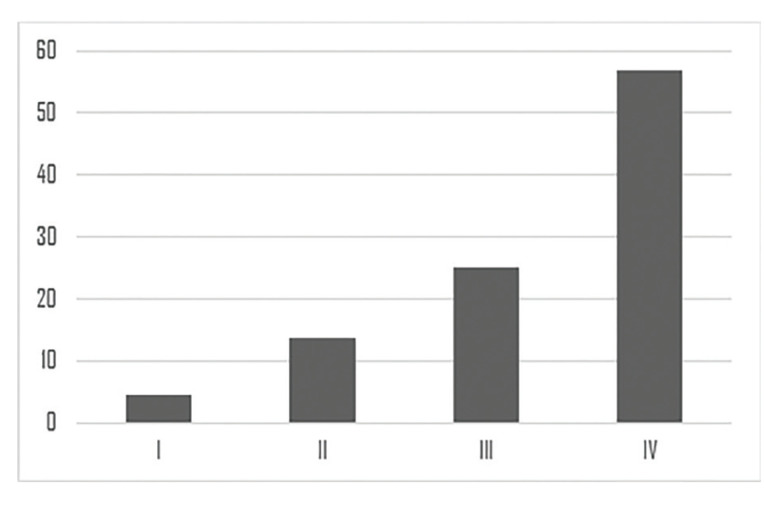
Sample distribution according to TNM staging.

Fig. 1 shows how HNC is primarily diagnosed in advanced stages, mainly in stages III and IV. Treatment included CX + RT in 25% (n = 43), RT + QT in 24.42% (n = 42), and CX + RT + QT in 18.60% (n = 32) (Table 2), with 78.95% (n = 45) receiving 60Gy or more.

- Prevalence of chronic oral complications

**Figure 2:**
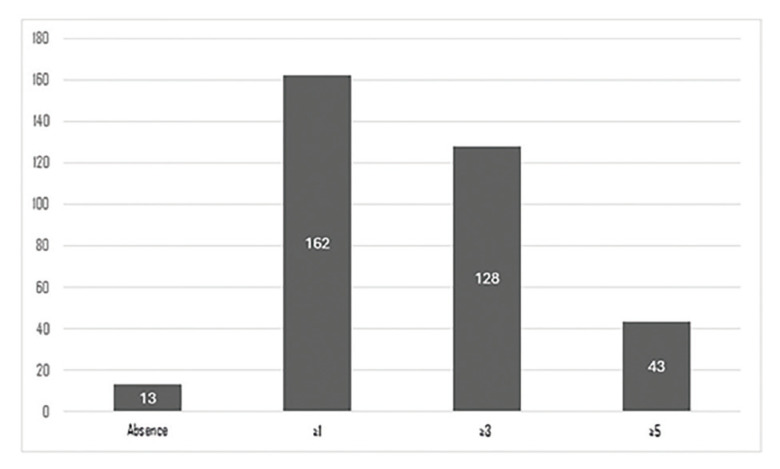
Sample distribution according to number of complications.

The average number of chronic oral complications in the total number of patients analyzed was 3.42 (SD = 1.92). In 76% of cases, the diagnosis of the alterations occurred more than six months after the end of treatment (n = 133), in 10.29% it was between three and six months later (n = 18), and in 13.71% it was less than three months (n = 24). During the follow-up, 97.75% (n = 87) showed a favourable evolution, with the majority (n = 75) exhibiting stabilization. The overall prevalence of complications for all treatments was 92.57% (n = 162). Fig. 2 reveals that 73.14% had 3 or more complications (n ​​= 128), and 24.57% had 5 or more (n = 43). Caries and periodontal disease were the most frequent, with prevalences of 81.14% (n = 142) and 65.14% (n = 114), respectively (Table 3).

- Risk indicators

No statistically significant association was observed with the prevalence or mean of chronic oral complications in terms of gender, number of cigarettes per day, tobacco use, alcohol consumption, oral hygiene, systemic pathology, active medication, invasive dental treatment, dental and periodontal status, histological type of cancer, and dose of RT (*p* > 0.05).

The average age of patients with oral complications was significantly higher than that of those without complications (*p* = 0.020). A remarkable association was observed regarding the location of the cancer (prevalence: *p* = 0.016; average: *p* = 0.037), showing a higher average value when it was found in the submandibular and parotid glands, lips, and floor of the mouth. Post hoc tests specifically revealed no differences in the number of alterations between locations. With reference to stages, there was no significant association with prevalence (*p* > 0.05), although appreciable differences were observed in the average (*p* = 0.023). Post hoc tests inferred significant differences only between stages II and III. Regarding oncological treatment, significant differences were shown in prevalence (*p* = 0.008), with 100% in multiple therapies. Additionally, noTable differences were identified between the averages of complications, being higher in options that combine multiple modalities (*p* < 0.001). Considering post hoc tests, relevant differences appeared specifically between CX versus CX + RT, RT + QT, CX + RT + QT, and CX + IT + RT + QT (Table 4). The study investigated associations between various treatments and specific complications. Hyposalivation (*p* < 0.001) and dental disease (*p* < 0.001) were significantly linked to combined treatments involving CX + RT, RT + QT, and CX + RT + QT. Chronic mucositis was notably associated with CX + RT, RT + QT, CX + RT + QT, and CX + IT + RT + QT (*p* = 0.017). Odynophagia showed significant associations with RT + QT and CX + RT + QT (*p* = 0.026). However, other complications studied did not significantly vary across different therapies.

Logistic regression analysis highlighted that advanced age, various tumor locations, advanced stages, and oncological treatment including RT did not independently associate significantly with complications. Variables initially identified as noTable risk factors did not maintain significance when adjusting for other factors concurrently. The study's findings suggested uncertainty in the estimated OR due to lack of significance and wide confidence intervals.

## Discussion

The overall prevalence was 92.57%. Wong *et al*. (5) indicated that between 90-100% of irradiated patients develop at least one complication. On the other hand, García *et al*. (21) documented acute QT complications in 86.99% of cases, and oral toxicity associated with IT and targeted therapies affects more than 20% (17), which are lower Figures compared to the other treatments.

The prevalence of hyposalivation/xerostomia was lower than reported in the literature, where OCSG (8) noted a range between 73.6-85.3% within one month to two years post-RT, with less involvement in intensity-modulated radiotherapy (IMRT). Others reported statistics of 75% (22) and 33.3% (23).

Taste disorders affect between 50-75% of patients or more during RT, with 15% remaining subsequently (5,14). However, the result of the study is lower compared to prevalences of 25% (23). On the other hand, dental disease was considerably higher than in other studies. Moore *et al*. (24) found that 37% developed caries due to radiation in the first two years, depending on the dose administered. Brennan *et al*. (25) observed an increase in the CAOS index with percentages ranging from 47.6% to 51.9% at various post-RT intervals. OCSG (11) indicated an average prevalence of 28.1%, and another research detected 16.6% (23). On the other hand, periodontal disease appeared in 65.14% of cases. Only one study had analyzed it in the long term, indicating a 50% active effect (23), and OCSG (11) established that 20.3% of QT patients showed gingivitis. Regarding pain, OCSG (9) reported 70% at the end of therapy, reducing to 36% after six months, a Figure that equates to the results obtained. Threet *et al*. (22) observed dental pain in 13% and 16% of oral burning four years later. Chronic mucositis showed a higher frequency compared to 8.1% at six months, with 9% in those receiving concurrent QT and 5.9% in those not (26).

Several reviews (5,6) report a range of 5-45% of trismus for those receiving RT, increasing with doses greater than 60 Gy. OCSG (10) reported 25.4% in conventional RT, only 5% in IMRT, and 30.7% in concurrent RT and QT. Threet *et al*. (22) observed trismus in 29%, and Lalla *et al*. (26) and Sollecito *et al*. (27) reported a significant reduction in maximum opening after six months.

No viral infection was documented, primarily because these usually occur in neutropenic patients with oral ulcerations undergoing QT, manifesting in 40% (5) and 49.8% (12). Clinically diagnosed fungal infections were lower than reported in the literature, which indicates approximately 30% (5,13) and odynophagia and dysphagia were also less frequent than in other studies, which report around 38% (22), with significant worsening of swallowing (26). On the other hand, ORN and OQN manifested within the framework established by the literature, between 4-8% (4,15,16). Regarding the type of treatment, 7.4% are described for conventional RT, 5.1% for IMRT, 6.8% for RT and QT, and 5.3% for brachytherapy (6).

Various alterations have been documented, highlighting the diversity of oral and systemic complications faced by oncological patients (17,23). However, for proper comparison between studies, there are differences in several aspects such as sample size, demographics, types of cancers, therapeutic regimens, methodology and measurement instruments, follow-up duration, and periodicity of assessments.

Regarding risk factors, the results suggest that the combination of factors such as advanced age, various locations, advanced stages of cancer, and oncological therapy with RT can significantly contribute to the development of chronic oral alterations. Several studies (18,19,21,28) state that smoking, HPV infection, and worse periodontal condition increase predisposition, as well as greater dental failure after therapy in patients with poorer initial oral condition (29). The risk of trismus increases with factors associated with cancer and treatment (10,27), and the predisposition to ORN increases mainly with irritant factors, invasive surgical treatments, and doses higher than 60Gy (6,15,16).

Knowledge of risk factors and the diversity of complications should facilitate the establishment of preventive, diagnostic, and therapeutic protocols in these patients, in order to optimize cancer care and minimize associated toxicity. The study provides a realistic view of clinical practice, allows examination of the individual and global impact of various treatments, provides knowledge about risk factors, and therefore helps identify susceptible groups. Additionally, it aids in designing customized programs. However, given the retrospective nature of the study, it must be assumed that there may be errors and confusions, absence of data, and lack of consensus in the medical histories analyzed, as well as not taking into consideration confounding variables that could influence the results. Furthermore, the follow-up of certain patients may have been inadequate, and the great diversity of variables analyzed has generated small samples in certain groups, which could lead to insufficient statistical power.

The high prevalence observed in the study makes diagnosis, and even more so, prevention crucial. The analysis of the different indicators allows for the risk stratification of cancer patients, and multidisciplinary education on specific preventive and therapeutic programs is a primary measure to improve the quality of life. Future research with prospective methodologies that address these limitations is required to more precisely study complications and their risk factors.

## Figures and Tables

**Table 1:** HNC location.

Location	n	%
Floor of the mouth	11	6.63
Tongue	37	22.29
Yugal mucosa	1	0.60
Gingiva	3	1.81
Palate	9	5.42
Mandible / Maxillary	27	16.27
Parotid	3	1.81
Submandibular	3	1.81
Pharynx	46	27.71
Larynx	23	13.86
Lip	3	1.81

**Table 2:** Oncological therapy.

Oncological therapy	n	%
CX	30	17.44
CX +RT	43	25
CX +QT	2	1.16
RT+QT	42	24.42
RT	7	4.07
QT	1	0.58
IT	1	0.58
IT+RT	1	0.58
CX +IT+RT+QT	7	4.07
CX+IT+RT	1	0.58
IT+RT+QT	3	1.74
CX+RT+QT	32	18.60
Other modalities	2	1.16

**Table 3:** Prevalence of chronic oral complications.

Chronic oral complications	n	%
Hyposalivation / Xerostomia	110	62.86
Taste dysfunction	22	12.57
Dental disease	142	81.14
Periodontal disease	114	65.14
Pain	60	34.29
Chronic mucositis	27	15.43
Trismus	23	13.14
Mucosal atrophy and fibrosis	24	13.71
Soft tissue necrosis	9	5.14
Viral infection	0	0
Fungal infection	16	9.14
Bacterial infection	10	5.71
Odynophagia	8	4.57
Dysphagia	17	9.71
ORN / OQN	13	7.43
Neuropathies	3	1.71

**Table 4:** Statistically significant associations between chronic oral complications and age, location, stage and oncological therapy.

Characteristics		Complications (n)		*p-value* (Fisher's exact test)	Mean	SD	*p-value* (Kruskal-Wallis)	*p-value* (Student's t-test)
		No	Yes					
Location	Floor of the mouth	0 (0,0%)	11 (100,0%)	0,016	3,73	1,62	0,037	-
	Tongue	3 (8,1%)	34 (91,9%)		3,65	2,14		
	Oral mucosa	1 (100,0%)	0 (0,0%)		0,00	--		
	Gingiva	0 (0,0%)	3 (100,0%)		2,33	1,16		
	Palate	2 (22,2%)	7 (77,8%)		3,33	2,83		
	Mandible / Maxillary	6 (22,2%)	21 (77,8%)		2,48	2,26		
	Parotid	0 (0,0%)	3 (100,0%)		4,67	1,16		
	Submandibular	0 (0,0%)	3 (100,0%)		5,67	2,52		
	Pharynx	1 (2,2%)	45 (97,8%)		3,61	1,54		
	Larynx	0 (0,0%)	23 (100,0%)		3,43	1,50		
	Lip	0 (0,0%)	3 (100,0%)		4,00	1,73		
Stage	I	0 (0,0%)	4 (100,0%)	0,063	2,75	0,50	0,023	-
	II	3 (25,0%)	9 (75,0%)		2,42	2,02		
	III	0 (0,0%)	22 (100,0%)		4,59	2,13		
	IV	3 (6,0%)	47 (94,0%)		3,80	1,85		
Oncological therapy	CX	6 (20,0%)	24 (80,0%)	0,008	1,77	1,28	<0,001	-
	CX + RT	3 (7,0%)	40 (93,0%)		3,74	2,03		
	CX + QT	0 (0,0%)	2 (100,0%)		3,00	0,00		
	RT + QT	0 (0,0%)	42 (100,0%)		3,76	1,43		
	RT	0 (0,0%)	7 (100,0%)		4,00	1,63		
	QT	0 (0,0%)	1 (100,0%)		3,00	--		
	IT	1 (100,0%)	0 (0,0%)		0,00	--		
	IT + RT	0 (0,0%)	1 (100,0%)		4,00	--		
	CX + IT + RT + QT	0 (0,0%)	7 (100,0%)		4,71	1,70		
	CX + IT+ RT	0 (0,0%)	1 (100,0%)		2,00	--		
	IT + RT + QT	0 (0,0%)	3 (100,0%)		3,33	0,58		
	CX + RT + QT	0 (0,0%)	32 (100,0%)		4,19	2,02		
	Other modalities	1 (50%)	1 (50%)		1,50	2,12		
Age	Absence complications	13 (7,4%)		-	55,79	13,70	-	0.020
	Presence complications	162 (92,6%)		-	63,53	11,22	-	
